# Complete mitochondrial genome of blunt slipper lobsters *Scyllarides squammosus* (H. Milne Edwards, 1837)

**DOI:** 10.1080/23802359.2019.1627935

**Published:** 2019-07-12

**Authors:** Hongtao Liu, Mingqiu Yang, Yugui He

**Affiliations:** Hainan Provincial Engineering Research Center for Tropical Sea-farming, Hainan Academy of Ocean and Fisheries Sciences, Haikou, China

**Keywords:** *Scyllarides squammosus*, mitochondrial genome, phylogenetic analysis

## Abstract

The complete mitochondrial genome of *Scyllarides squammosus* was first determined and characterized. With a length of 15,644 bp, it consists of 22 tRNA genes, 2 rRNA genes, 13 protein-coding genes (PCGs), and 1 control region. The nucleotide composition is significantly biased with AT contents of 65.6%. Among these PCGs, five of them used an unusual initiation codon, and nine genes ended with an incomplete or abnormal stop codon. Two microsatellites were identified and located in *COX3* gene and D-loop region. Phylogenetic analysis demonstrated that *S. squammosus* was first clustered with *Scyllarides latus*, which was consistent with the previous work.

*Scyllarides squammosus*, known as scaly slipper lobster or blunt slipper lobster, is a highly valuable species in the family Scyllaridae, Achelata, the price of which is almost similar to lobster due to scarce and delicacy. *S. squammosus* habits on reefs and rocky areas commonly at depth of 7.5–71 m. It has an extensive distribution in the Indo-West Pacific region: from East Africa to Japan, Hawaii, Melanesia, New Caledonia, and Australia. Till now, many researchers have long studied the biology of *S. squammosus* such as feeding behavior (Lau 1987), fecundity and egg size (DeMartini and Williams 2001), sexual maturity (DeMartini et al. 2005), gamete and larval development (Matthews 1954; Coutures 2001), fishery (Clarke and Yoshimoto 1990; O'Malley 2004), movement pattern (O'Malley and Walsh 2013) and Spatiotemporal variation on the population ecology (O’Malley 2011).

The samples were collected from Huanqiu wharf of Wenchang, Hainan province, China (19°33′57.91″ N, 110°49′12.10″ E), and stored in Qionghai research base of Hainan Academy of Ocean and Fisheries Sciences for reference and DNA extraction.

The complete mitogenome sequence of *S. squammosus* is 15,644 bp in length (GenBank Accession no. MK783265). The base content was 31.2% A, 13.2% G, 34.3% T, and 21.2% C. The 65.6% of (A + T) showed great preference to AT. the circular mitogenome contained 22 tRNA genes, 2 rRNA genes, 13 protein-coding genes (PCGs), and 1 control region (D-loop). Four PCGs (*ND1*, *ND4*, *ND4L*, and *ND5*), eight tRNA genes and two rRNA genes were encoded on the light strand, the others were encoded by the heavy strand.

The 22 tRNA genes of the *S. squammosus* mitogenome ranged in length from 63 to 73 bp. The genes *tRNA-Leu* and *tRNA-Ser* have two copies each, identified with different codons (*tRNA-leu* uses TAA and TAG; *tRNA-Ser* uses TCT and TGA). The 12S rRNA is located between *tRNA-Val* and D-loop with the length of 863 bp, and the 16S rRNA is 1339 bp, located between *tRNA-Val* and *tRNA-Leu*. Except for five PCGs using an abnormal start codon (*ND1* and *ND4L* use TTA; *COX1* uses ACG; *ND4* uses CAG; *ND5* uses AAC), the others use a common initiation codon ATN. We also found that except for 4 PCGs using TAA or TAG, the stop codon of the other nine genes were abnormal: *COX2*, *ND2*, *ND3*, and *ND5* use a single base T; *ND4* uses AT; *COX1* uses TT; *CYTB* uses TG; *ND4L* uses CAT; and *ND1* uses CAC. With a length of 716 bp, the control region is located between 12S rRNA and *tRNA-Ile*. Two microsatellites (SSR) were found in the mitogenome of *S. squammosus* using MISA software. The two (T)_10_ SSRs were located in the codon region of *COX3* gene and the non-codon region of D-loop, respectively.

To investigate the phylogenetic relationship of *S. squammosus* in the Achelata, a phylogenetic tree was constructed based on the13 PCGs nucleotide sequences of 16 Achelata species mitogenome available in the GenBank using the maximum-likelihood (ML) method with 1000 bootstrap replicates. The result (Figure 1) show that *S. squammosus* was first clustered with *Scyllarides latus*, which was consistent with the previous work (Palero et al. 2016).

**Figure 1.**
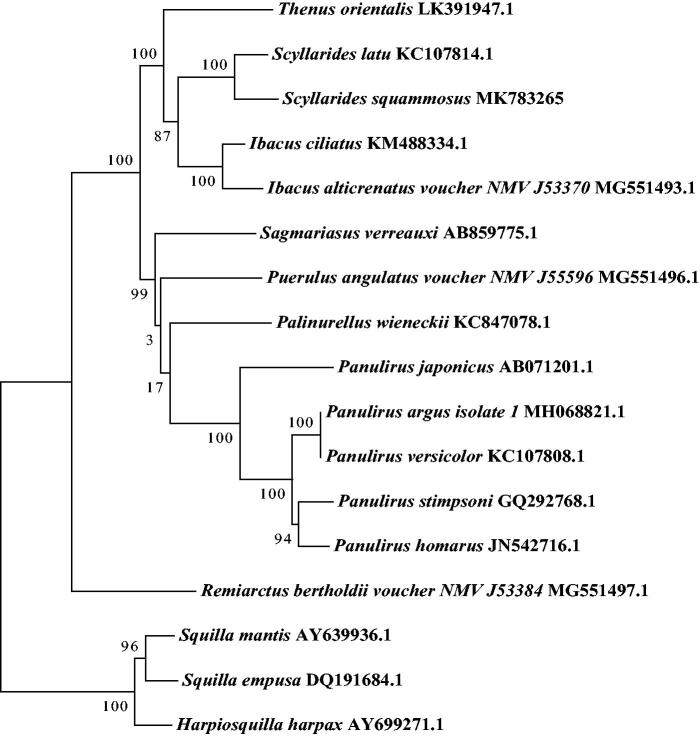
Phylogenetic tree of the complete mitogenome of 16 species in Achelata. *Harpiosquilla harpax*, *Squilla empuse*, *and Squilla mantis* were used as outgroups.
